# Phytochemical‐Based Strategies for Lung Cancer: Clinical Insights Into Pleiotropic Molecular Signaling and Therapeutic Roles

**DOI:** 10.1002/ptr.70250

**Published:** 2026-02-04

**Authors:** Md. Rezaul Islam, Abdur Rauf, Happy Akter, Md. Ibrahim Khalil Al‐Imran, Md. Naeem Hossain Fakir, Gazi Kaifeara Thufa, Umme Habiba, Karjin Nahar Riya, Md. Mahfuzur Rahman, Md Sadique Hussain, Hanan A. Ogaly, Abdullah S. M. Aljohani, Waleed Al Abdulmonem, Dorota Formanowicz, Marcello Iriti

**Affiliations:** ^1^ Department of Pharmacy, Faculty of Health and Life Sciences Daffodil International University Dhaka Bangladesh; ^2^ Department of Chemistry University of Swabi Anbar Khyber Pakhtunkhwa Pakistan; ^3^ Padma View College of Nursing Dhaka Bangladesh; ^4^ Uttaranchal Institute of Pharmaceutical Sciences, Uttaranchal University Dehradun Uttarakhand India; ^5^ Chemistry Department College of Science, King Khalid University Abha Saudi Arabia; ^6^ Department of Medical Biosciences College of Veterinary Medicine, Qassim University Buraydah Saudi Arabia; ^7^ Department of Pathology College of Medicine, Qassim University Buraydah Saudi Arabia; ^8^ Chair and Department of Medical Chemistry and Laboratory Medicine Poznan University of Medical Sciences Poznan Poland; ^9^ Department of Biomedical, Surgical and Dental Sciences University of Milan Milan Italy; ^10^ National Interuniversity Consortium of Materials Science and Technology (INSTM) Firenze Italy

**Keywords:** lung cancer, molecular signaling pathways, phytochemicals, prevention, targeted therapy, treatment strategies

## Abstract

Lung cancer (LC) remains the leading cause of global cancer‐related death due to delayed diagnosis, poor therapeutic efficacy, and drug resistance. Traditional therapeutic methods like radiation, chemotherapy, and targeted medicines are often associated with high toxicity and often result in minimal survival improvements. Phytochemicals from medicinal plants are increasingly being considered as potential LC treatment agents due to their multi‐targeted action, safety, and accessibility. These have anticancer properties by regulating key molecular signaling pathways like PI3K/Akt/mTOR, MAPK/ERK, NF‐κB, STAT3, and apoptotic cascades. These compounds also promote apoptosis, increase chemotherapeutic medication sensitivity, and prevent tumor cell growth, angiogenesis, invasion, and metastasis. Phytochemicals have shown potential in reducing therapy‐induced side effects and combating multidrug resistance, potentially enhancing treatment effectiveness. Despite promising discoveries, challenges such as low bioavailability, limited pharmacokinetic stability, and lack of extensive clinical validation inhibit their widespread use. This review provides clinical insights into phytochemical‐based LC preventive and treatment approaches, focusing on their role in addressing molecular signaling pathways. It demonstrates the potential medicinal benefits, potential disadvantages, and potential applications of phytocompounds as supplementary or alternative treatments for LC.

## Graphical abstract

Lung cancer (LC) remains the leading cause of global cancer‐related death. Phytochemicals from medicinal plants are increasingly being considered as potential LC treatment agents due to their multi‐targeted action, safety, and accessibility. These have anticancer properties by regulating key molecular signaling pathways.

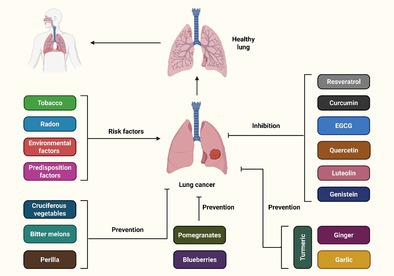

## 1 Introduction

**FIGURE 1:**
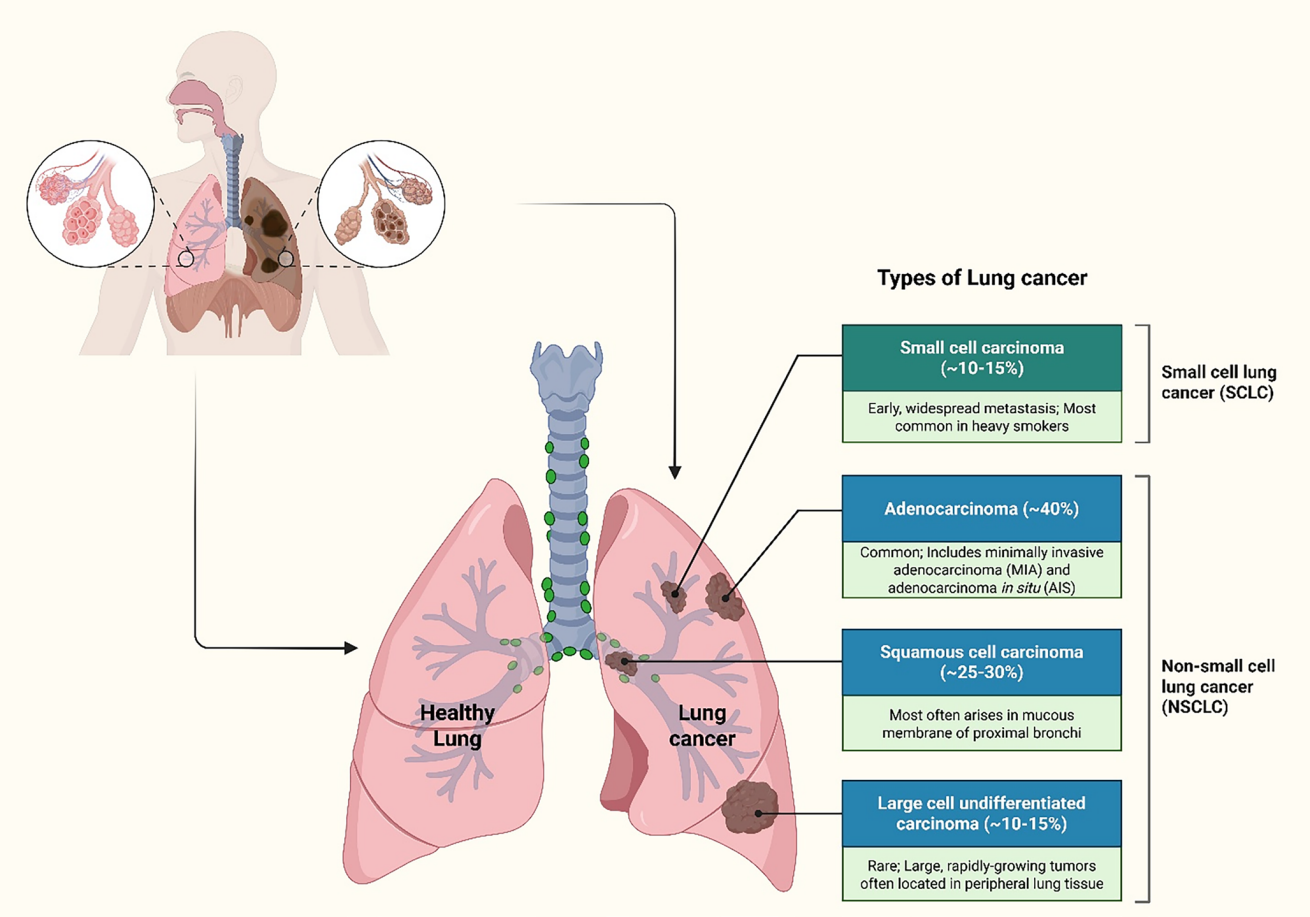
The two primary types of lung cancer are small‐cell lung cancer (SCLC) and non‐small‐cell lung cancer (NSCLC).

Lung cancer (LC) has the highest fatality rate among cancer‐related causes of death (Babar et al. 2020). Additionally, LC is the second most prevalent tumor worldwide, with 19,292,789 new cases reported in 2020, with 14.3% of cases in men. It ranks as the world's largest cause of cancer‐related fatalities, with 1,796,144 recorded deaths (Ferlay et al. 2019). LC (Figure 1) is the most prevalent cancer and a major contributor to cancer‐related mortality, with approximately 2 million new diagnoses and 176 million deaths worldwide (Shen et al. 2023). According to GLOBOCA statistics, in 2022, LC was the most frequently diagnosed cancer in men, with 1.57 million new cases reported. LC was the second most diagnosed cancer in women, with 0.91 million new cases reported. It is the leading cause of cancer death globally, accounting for 1.23 million deaths (Filho et al. 2025). Natural compounds, known for their medicinal properties and low toxicity, have the potential role in LC prevention and treatment (Jiao et al. 2016; Liu et al. 2018). Furthermore, natural compounds have few adverse effects and show potential efficacy in LC treatment. When combined with anti‐cancer medications, they form a synergistic effect that enhances therapeutic outcomes. Cancer dysregulates apoptosis, allowing survival and spread of cancer cells. Natural substances can selectively target specific cell signaling pathways, inducing cytotoxicity in cancer cells (Al‐Yozbaki et al. 2021). Research indicates that diets rich in fruits and vegetables, rich in natural products, reduce the risk of LC development. These exhibit antioxidant and anti‐inflammatory properties, effectively combating oxidative stress and chronic inflammation, which are key contributors to LC initiation and promotion (Abdulla and Gruber 2000; Baena Ruiz and Salinas Hernández 2016; Darvesh and Bishayee 2013; Liu et al. 2020; Liu 2003, 2004; Rodriguez‐Casado 2016). Their mechanisms of action involve targeting various signaling pathways and specific molecules that promote angiogenesis, invasion, and proliferation of cancer cells (Aggarwal et al. 2007; Pratheeshkumar et al. 2012). Natural compounds demonstrate significant anticancer effects against LC by inhibiting A549 cell growth in a dose‐dependent manner. These compounds influence proteins involved in the cell cycle and apoptosis, affecting B‐cell lymphoma‐2 and poly(ADP‐ribose) polymerase expression. Additionally, these compounds also promote autophagy and cause cell cycle arrest at the S and G2/M phases (Lin et al. 2019). This review explores the preventive and therapeutic functions of phytochemicals in LC treatment.

## 2 Etiology of LC

LC is the leading cause of cancer‐related deaths among women in the US and Western countries, with active cigarette smoking being the leading cause. However, there are differences in the disease's etiology between men and women, such as women being more likely to develop LC among never smokers, estrogen potentially increasing the risk, and women having a higher proportion of adenocarcinomas and better prognosis (Alberg et al. 2013). One known risk factor for LC is cigarette smoking (Cancer 2004). The International Agency for Research on Cancer (IARC) has identified over 70 carcinogens in cigarettes as human carcinogens, and IARC monographs have compiled epidemiologic studies showing a link between smoking cigarettes and LC (Cancer 2004; Humans 2012). The risk of LC rises with the length and intensity of cigarette smoking, and present smokers are more at risk than former smokers. Differences in smoking initiation age, smoking duration, cigarette filters, tobacco product ingredients, and other environmental and lifestyle factors may account for some of the racial and ethnic differences in the risk of LC found in other studies (Humans 2012). Whites and African Americans were among those who smoked no more than 20 cigarettes per day, while Japanese Americans and Latinos had the lowest risk. These racial and ethnic differences ceased to be significant among those who smoked more than 30 cigarettes per day (Haiman et al. 2006). Cigarette smoking is strongly linked to the development of LC, with increased risk correlated to the duration and severity of smoking.

## 3 Risk Factors of LC

### 3.1 Tobacco

**FIGURE 2:**
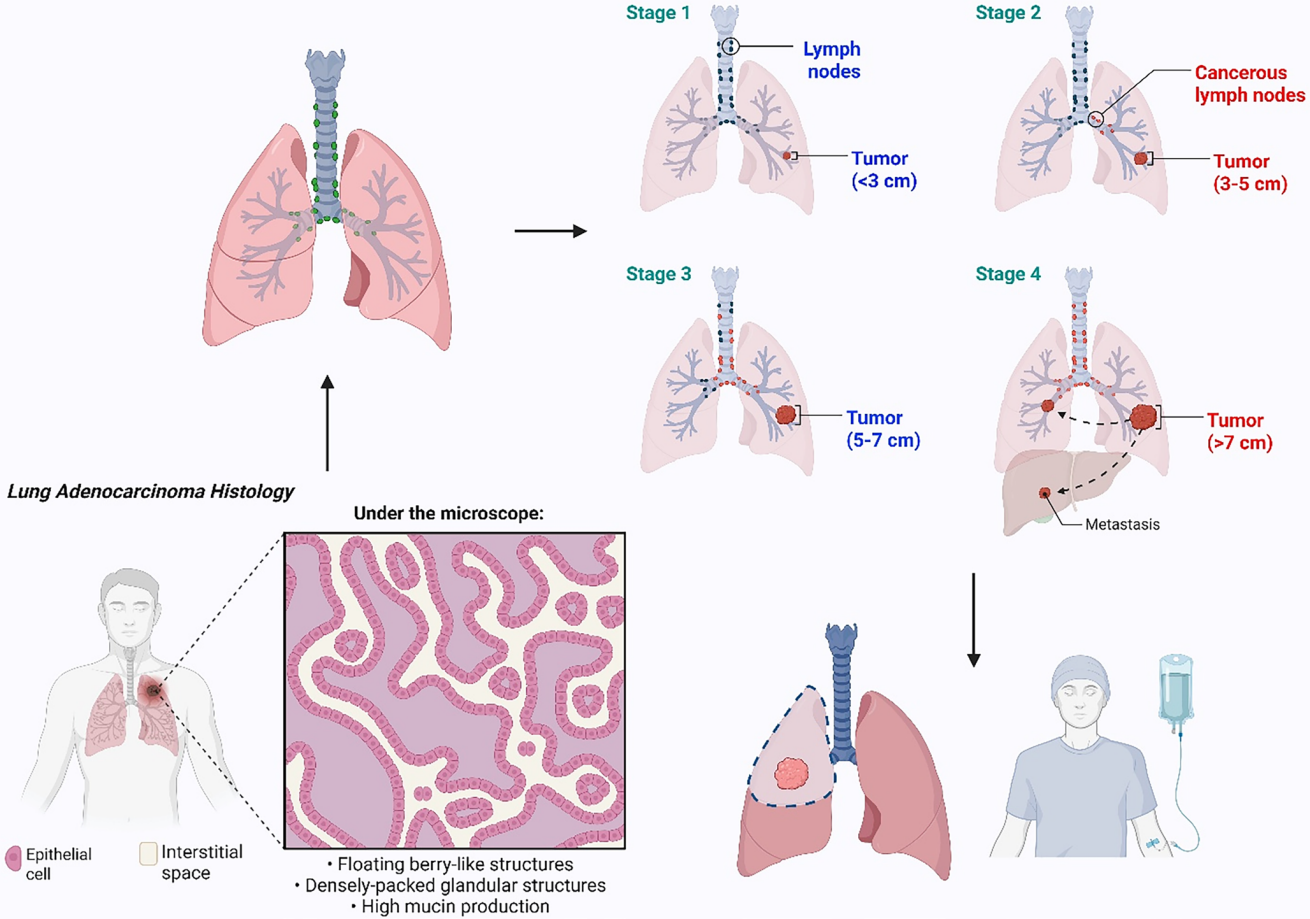
The histology of lung adenocarcinoma and its stages. The stages of lung cancer consist of four stages, such as Stages 1, 2, 3, and 4. Stage 1 cancer is non‐expanding, Stage 2 is larger, has lymph nodes within the lung, and multiple tumors in the same lobe, Stage 3 is larger, and has nearby lymph nodes or structures, and Stage 4 spreads to distant organs.

Smoking‐related causes resulted in 1.7 million deaths worldwide, with LC accounting for a significant portion of cancer‐related fatalities (Hotez et al. 2016). Smoking is the primary cause of LC deaths, responsible for over 70% in men and 55% in women (Hotez et al. 2016). Tobacco use rates vary significantly among communities, with women in Asian and African nations having rates as low as 5% and males as high as 40% (Reitsma et al. 2017). Smoking is the main risk factor for LC, responsible for 75%–80% of deaths in women and 90% in men annually in the US (Boyle 1997; Shopland 1995). Additionally, smoking cigarettes is linked to LC due to the carcinogenic polycyclic aromatic hydrocarbons in tobacco smoke, which modify the p53 gene, promoting cancer progression (Pfeifer et al. 2002). Smoking‐related LC is a molecular biomarker for tobacco‐induced alterations, linked to G to T transversions in the gene. N‐nitroso compounds, found in tobacco smoke, contain animal carcinogens (Vineis et al. 2004). Lung adenocarcinoma incidence has increased more rapidly in both men and women in recent decades than squamous cell carcinoma incidence (Stellman et al. 1997). Modern cigarettes emit smoke with higher nitrosamine levels, which favorably raise the incidence of adenocarcinoma relative to other cell types (Stellman et al. 1997). Adenocarcinoma (Figure 2), once the most prevalent histologic type of LC, has increased in prevalence from 25% to 33% in males due to a decrease in tars and nitrosamines (Harkness et al. 2002; Patel and Obrams 1995). Smoking cigarettes with a medium, low, or extremely low tar content all carry the same risk of developing LC (Harris et al. 2004). Additionally, smoking can increase the risk of adenocarcinoma and cancer‐causing substances (Benowitz 2001). A study demonstrates a sex‐specific relationship between smoking and LC (Yu et al. 2014). Tobacco carcinogens cause genetic mutations, particularly in p53, leading to a higher incidence of adenocarcinoma.

### 3.2 Radon

Radon is a primary risk factor for LC in nonsmokers and smokers, with radon‐prone regions in Europe. It damages DNA and increases genomic tumor instability (Riudavets et al. 2022). A study investigated radon‐induced LC in rats exposed to high radon concentrations. It found frequent deletions in human chromosome homologs, including tumor suppressor genes and proto‐oncogenes (Dano et al. 2000). Another study found dysregulation of the INK4a/CDK4/RB1 pathway in radon‐induced LC in rats, suggesting an underlying carcinogenic process (Bastide et al. 2009). Furthermore, a study demonstrates tumors with high radon exposure have higher levels of genes related to DNA damage and repair, including TP53, ATR, ATRX, BARD1, RAD50, and SMARCA4 (Lim et al. 2019). Radon exposure can cause genetic mutations and chromosomal disruptions, interfering with the cell cycle and the regulation of proteins and cytokines associated with cancer development. Research on uranium miners indicates that investigating LC tissues and peripheral lymphocytes provides substantial evidence supporting the carcinogenic effects of radon. Specific TP53 polymorphisms and mutations are linked to tobacco smoke (Popp et al. 1999; Vähäkangas et al. 2001). Radon significantly increases the risk of LC, particularly in nonsmokers, by causing DNA damage, genomic instability, and mutations in tumor suppressor genes.

### 3.3 Environmental Factors

Never‐smokers estimate slightly more than half of LC. The molecular profile and responsiveness to targeted therapy differ between non‐smokers and smokers. Identifying genetic and environmental variables contributing to LC in nonsmokers is crucial. Exposure to carcinogens like radon and secondhand tobacco smoke is strongly associated with non‐smoking LC (Samet et al. 2009). Exposure to hazardous pesticides, industrial chemicals, and aflatoxin in the environment can contribute to cancer development. Agriculture and public health workers are most exposed to these chemicals, with some LC incidences linked to their use. The occupational hazard ratio is higher in high exposure categories (Bonner et al. 2017). A study found a significant link between pesticide use and LC incidence, indicating that pesticides contribute to the development of LC (Alavanja et al. 2004). LC in humans is linked to tobacco use. Smoking for longer periods and more cigarettes increases the risk of LC death. Secondhand or passive tobacco smoke exposure is linked to LC (Butler et al. 2017; Sun et al. 2017). Non‐smoker LC is significantly associated with environmental factors, including radon, secondhand smoke, pesticides, and industrial chemicals.

### 3.4 Predisposition Factors

Lung carcinogenesis involves genetic abnormalities and epigenetic changes, influenced by aberrant gene function and transcriptional silence caused by CpG island hypermethylation. Research on humans and animals has revealed the role of gene‐specific methylation, exposure to carcinogens, and pharmacologic agents in cancer prevention (Piperi et al. 2008). A study involving four Nordic twin cohorts found that smoking status significantly influences the likelihood of developing LC. The relative recurrence risk ratio for LC in ever‐smoking pairs dropped with age, while for non‐smoking pairs it remained constant. Age reduces the contribution of familial effects, and smoking is the cause of LC (Hjelmborg et al. 2017). Tobacco use is the main risk factor for 90% of LC cases. Other factors include genetic predisposition, environmental pollution, occupational exposures, and infectious diseases. Clinical and molecular characterization helps differentiate subtypes, improve patient outcomes, and customize treatment for different communities (Laguna et al. 2024). Another study found that individuals with p53 germline mutations have a higher risk of LC. The researchers evaluated the incidence of lung and smoking‐associated malignancies in 1230 noncarriers and 33 carriers of germline p53 mutations. Smoking mutation carriers were 3.16 times more likely to develop LC than nonsmoking mutation carriers. The study develops early diagnosis and treatment methods for smoking‐related and lung malignancies (Hwang et al. 2003). Smoking is the main risk factor for LC, driven by genetic mutations and epigenetic changes, with environmental factors and genetic predispositions also contributing.

## 4 Mechanisms

### 4.1 Topoisomerase Catalytic Suppression or Topoisomerase Toxicity

Antineoplastic drugs frequently target DNA topoisomerase II in LC treatment (Larsen et al. 2003). Topoisomerases are essential enzymes involved in DNA replication and cell division, as they use torsional pressures to cleave and separate DNA strands (Eckardt et al. 1995). Additionally, topoisomerase‐DNA complex formation is a reversible process, primarily occurring in rapidly dividing cells such as cancer cells. Inhibiting topoisomerase activity can delay this process (Schmidt et al. 2012). Topoisomerases break the DNA double helix, with type I splitting one DNA strand and type II splitting two DNA strands (Froelich‐Ammon and Osheroff 1995). Moreover, topoisomerase‐targeting medications are categorized into topoisomerase inhibitors and topoisomerase synergist inhibitors, which inhibit topoisomerase activity by forming irreversible topoisomerase‐DNA complexes (Berger et al. 1998). Furthermore, topoisomerase poisons can intercalate into DNA strands, while intercalating medications like doxorubicin, amsacrine, ellipticine, and protoberberines, and non‐intercalating medications like etoposide and teniposide (Denny and Baguley 2003; Ratain and Rowley 1992). Etoposide, a dangerous topoisomerase II poisonous compound, and lycobetaine, an anti‐cancer alkaloid, reduce lung breakdown in mice (Bailly 2003; Kamal et al. 2015; Tang et al. 2003). Lycobetaine (IC_50_ = 1.2 mM) inhibited the growth of the LXFL529L cell line (Barthelmes et al. 2001), and was given to naked mice with large cell LC LXFL529 at a dose of mg/kg intraperitoneally on Days 1–4 and 8–12, significantly slowing the growth of the tumors (Barthelmes et al. 2001). Lycobetaine inhibited topoisomerases I and II, slowed cell development, stabilized the DNA‐topoisomerase I complex, and activated passing (Tang et al. 2003). Additionally, lycobetaine is a specific topoisomerase IIB poison in extra cancer cells (Barthelmes et al. 2001). Lycobetaine is an intercalating agent that impacts DNA base pairs, particularly the GC pair, through its interactions (guanine‐cytosine) (Liu et al. 1989; Tang et al. 2003; Wang et al. 1996). Moreover, lycobetaine's anticancer properties are attributed to its betaine and methylenedioxy groups, while its quaternary nitrogen particle is crucial for hydrogen connections with the oligonucleotide (Eckardt et al. 1995). Nitidine, a DNA intercalator, exhibits anticancer effects against LC in mice; however, its required dosages may be higher than anticipated, risking damage to topoisomerase II (Fleury et al. 2000; Tang et al. 2003; Wang et al. 1993). *Anonaceous acetogenins* exhibit cytotoxicity and anticancer properties, with gigantetrocin A, B, and annonacin showing strong LC cell cytotoxicity, but less effective against Meth‐A cells than Adriamycin (Wang et al. 2001). Inhibitors like etoposide and lycobetaine disrupt DNA replication and promote tumor suppression, identifying topoisomerases as crucial targets for LC treatment.

### 4.2 Prevention of DNA Synthesis

Reducing DNA synthesis reduces cell division, promoting anticancer action. However, other pathways, such as topoisomerase inhibitors, alkylating drugs, tablets, and antimetabolites, inhibit DNA synthesis, thereby impacting various cell division processes (Brunello et al. 2008; Rabindran et al. 2004; Thirumaran et al. 2007). Specific DNA polymerase inhibitors like aphidicolin are utilized in experimental pharmacology and biochemistry (Byun et al. 2005; Holm et al. 1989). Resveratrol was administered intraperitoneally at 2.5 mg/day, and it has been demonstrated that 10 mg/kg (but not 0.6 mg/kg) has chemopreventive and anticancer effects against LC (Athar et al. 2007). Additionally, resveratrol significantly reduced tumor weight and volume in metastatic LC in mice by 44% and 42%, primarily due to inhibition of VEGF and apoptosis in LC cells (Ko et al. 2017; Vervandier‐Fasseur and Latruffe 2019). Indirubin has been linked to anti‐leukemic activity, as it inhibited leukemia cell death in mice (Cheng et al. 2017). Furthermore, indirubin inhibits DNA polymerase I and displays anticancer properties by preventing DNA synthesis, as evidenced in various cell lines and in living rats with Walker 256 sarcoma (Ancuceanu and Istudor 2004; Eisenbrand et al. 2004). Proanthocyanidin extract inhibits LC cells in vitro in A‐427 human LC cells (Gilbert et al. 1995; Riou et al. 1992). Ursolic acid showed strong inhibitory effects on human DNA topoisomerases I and II, DNA polymerase alpha and beta in calf and rat, but did not function as a topoisomerase toxin (Barthomeuf et al. 2002). Additionally, ursolic acid induces apoptosis in A549 cell lines, but has a negligible effect on H460 cell lines (Park et al. 2001). The *Oldenlandia diffusa* extract showed lower cytotoxicity toward healthy BEAS‐2 lung epithelial cells, but cytotoxic effects on H69 and H69VP LC cells via apoptotic pathways (Choi et al. 2000). LC cell proliferation is inhibited by blocking DNA synthesis using DNA polymerase inhibitors, alkylating drugs, and topoisomerase inhibitors.

### 4.3 Suppression of Protein Synthesis

Lincosamides, antibiotics that inhibit protein synthesis, are not commonly used in cancer treatment. However, certain substances like mistletoe lectins, procainamide‐type lignans, alkaloids, and quassinoids have shown anticancer efficacy (Baumann et al. 2002; Hiort et al. 1999). The rocaglamide lignans can inhibit protein synthesis without affecting nucleic acid synthesis (Kinghorn et al. 2003; Lee et al. 1998). Lignanans showed potent cytotoxic activity against the A549 LC cell line and cancer cell lines (Wang and Duh 2001). Independent studies have confirmed the effects of rocaglamide lignans on protein synthesis and cytotoxicity in various cancer cell lines (Bohnenstengel et al. 1999; Ohse et al. 1996). Protein synthesis inhibitors like rocaglamide lignans, mistletoe lectins, and specific alkaloids exhibit cytotoxic effects on LC cells.

### 4.4 Modulation of Lipoxygenase Pathways

Arachidonic acid, when converted by lipoxygenases, forms leukotrienes or prostanoids, which in humans and animals inhibit tumor development (Levin and Marnett 1994; Marnett 1992). The release of arachidonic acid from membrane phospholipids significantly contributes to cancer cell growth, and inhibiting lipoxygenase enzymes may reduce or prevent tumor formation (Wong et al. 2001). Baicalein exhibits inhibition of 12‐lipoxygenase and has been investigated for its potential in treating various malignancies. It has been found to induce apoptosis in the gastric (Wong et al. 2001), pancreatic (Tong, Ding, Witt, and Adrian 2002), human breast (Tong, Ding, and Adrian 2002), and prostate cancer cells (Pidgeon et al. 2002). Furthermore, baicalein may affect LC and significantly suppress topoisomerase II in various tumor cells, including human hepatocellular carcinoma (Austin et al. 1992; Matsuzaki et al. 1996). Additionally, baicalein can block topoisomerase I (Tang et al. 2003). Baicalein inhibits matrix metalloproteinases to induce antiangiogenic effects (Wartenberg et al. 2003). Wogonin, a cancer cell suppressor, induces apoptosis, activating caspase 3 and producing p53 and p21 proteins, but its ability inhibits 12‐lipoxygenase (Chen et al. 2002; Lee et al. 2002).

## 5 The Molecular Basis of Phytocompounds in the Treatment of LC

The role of phytocompounds in the prevention and treatment of LC. The various types of phytocompounds show anticancer activity against LC based on molecular mechanisms (Table 1).

**TABLE 1:** A list of phytocompounds that may have anticancer properties against different types of LC cell lines.

Phytocompounds	Concentration	Cell line	Mechanism of action	Major finding	References
Genistein	20–40 μM	SPC‐A‐1	Cell cycle arrest, antiproliferation, and apoptosis induction by control of apoptosis‐related genes	Reduced the viability of human lung adenocarcinoma SPC‐A‐1 cells, causing cell cycle arrest and apoptosis.	(Zou et al. 2008)
Phloretin	125–150 μg/mL	A549 Calu‐1 H838 H520	Reduced growth, apoptosis induction, inhibition of Bcl‐2 expression, and elevated production of cleaved caspase‐3	Enhanced the anticancer effects of cisplatin, suggesting a potential combination therapy for NSCLC treatment.	(Ma et al. 2016)
EGCG	5–50 μM	H1299 H460	Enhanced miR‐210 expression, which inhibits growth	Upregulated miR‐210 expression in LC cells, reducing cell proliferation and sensitivity to EGCG.	(Wang et al. 2011)
Quercetin	0.74–4.40 μmol/L	A549	Dose‐dependent reduction in cell proliferation and enhancement in apoptosis	The combination inhibited cancer cell growth and induced apoptosis, triggering microtubule alteration, and increased G2/M phase cells	(Zhang, Wang, et al. 2015)
Luteolin	20–80 μM	A549 H460	Reduction in cell division by Tyro3, Axl, and MerTK (TAM) receptor tyrosine kinases (RTK) downregulation	Luteolin has cytotoxic effects on NSCLC cells, including cisplatin‐resistant ones, and decreases clonogenic ability.	(Lee et al. 2017)
Ferulic acid	50–1000 μM	A549	Reduced cell adhesion, migration, proliferation, and formation of superoxide anion	It is used in the prevention and treatment of LC.	(Bouzaiene et al. 2015)
Gallic acid	5 μM	H1993	Suppression of Bcl2 and cyclin D, two STAT3 target genes that are downregulated and cause apoptosis and cell cycle arrest.	It has potential adjuvants to overcome tyrosine kinase inhibitor resistance in advanced LC.	(Jeong et al. 2017)
Curcumin	10–20 μM	A549	The suppression of invasion and metastasis was mediated by MMP.	Inhibited human LC cell migration and invasion in vitro.	(Lin et al. 2009)
Resveratrol	4–64 μM	A549	Growth inhibition and apoptosis triggered by activation of caspase 3	Resveratrol has been shown in a xenograft model using A549 cells to have the ability to suppress the growth of LC cells both in vitro and in vivo.	(Yin et al. 2013)
Isorhamnetin	16 μM	A549	Inhibition of colony formation and cellular proliferation, along with an increase in apoptosis through the activation of caspase in the mitochondria‐dependent pathway	Inhibited human LC A549 cells by suppressing proliferation and colony formation, causing apoptosis, and promoting autophagosome formation.	(Ruan et al. 2015)
Kaempferol	10–140 μM	A549	Antiproliferative action that is dose‐dependent and reduces metastasis by inhibiting EMT	Increased E‐cadherin expression in A549 LC cells inhibits the EMT.	(Hang et al. 2015)
Hesperidin	5–50 μM	A549 NCIH358	Inhibited proliferation and apoptosis by reducing mitochondrial membrane potential.	Showed anti‐proliferative and pro‐apoptotic effects in NSCLC cells.	(Birsu Cincin et al. 2015)

### 5.1 Resveratrol

Plants such as grapes, peanuts, mulberries, and legumes contain phytoalexin resveratrol (Aggarwal et al. 2004). The anti‐proliferative effects of resveratrol on LC have led to advancements in treatment. It inhibits cell division, initiates programmed cell death, and stops the cell cycle (Whyte et al. 2007). The anticancer properties of resveratrol involve promoting early senescence in LC cells, resulting in increased DNA double‐strand breaks and the generation of ROS (Luo, Wang, et al. 2013; Luo, Yang, et al. 2013). Resveratrol‐induced early senescence in LC cells is associated with higher ROS and double‐strand breaks (Luo, Yang, et al. 2013). Additionally, resveratrol enhances the efficacy of gefitinib, an EGFR inhibitor, in human NSCLC cell lines, regardless of mutation status, by increasing sensitivity and enhancing its inhibitory effect (Zhu et al. 2015). Resveratrol exerts its anticancer effects in LC through the modulation of microRNAs (miRNAs) (Bae et al. 2011; Han et al. 2012; Yu et al. 2013). Furthermore, resveratrol treatment in LC cells alters the expression levels of various miRNAs, including those affecting cell differentiation, proliferation, cell cycle control, and apoptosis (Bae et al. 2011). Resveratrol‐induced elevated miR‐622 levels have been linked to decreased colony formation, cell proliferation, and tumor development due to its suppression of K‐Ras's expression (Han et al. 2012). Resveratrol inhibits LC cell proliferation through apoptosis induction, cell cycle arrest, and ROS‐mediated DNA damage.

### 5.2 Curcumin

Curcumin therapy during the G2/M phase resulted in cell cycle arrest in H460 cells. The cell cycle underwent several molecular changes, including the overexpression of Bax, Bad, and FAS/CD95, and the downregulation of cyclin D and E. Additionally, the proteins Bcl‐2, Bcl‐xL, and XIAP are downregulated. Curcumin therapy elevated endoplasmic reticulum stress, intracellular Ca^2+^, and ROS, causing decreased CDK1, mitochondrial membrane potential reduction, growth arrest relaxation, caspase‐3 activation, GADD153 and GRP78 transfer, and increased endo G mRNA expression (Wu et al. 2010). Additionally, curcumin reduced anti‐apoptotic Bcl‐2 expression in A549 cells, promoting pro‐apoptotic Bax expression, leading to mitochondrial apoptosis and reduced cell proliferation (Li et al. 2013). Curcumin inhibited PC‐9 cell growth and caused cell cycle arrest during the G1/S phase, enhancing gene expression and downregulating essential cell cycle genes (Saha et al. 2010). A501, a synthetic curcumin derivative, exhibits potent anticancer properties by inhibiting cyclin B1 and cdc‐2 expression, causing cell cycle arrest, and promoting apoptosis through Bax and p53 expression (Xia et al. 2014). The effect of curcumin on gene expression, including GLUT1, MT1‐MMP, and MMP2, inhibits A549 cell proliferation, invasion, and metastasis, with MMP2 found in these cells (Liao et al. 2015). Curcumin reduces tumor cell invasion and metastasis in vitro and vivo, modulating the GLUT1/MT1‐MMP/MMP2 pathway. Overexpression of GLUT1 may cause resistance to curcumin treatment in LC patients (Liao et al. 2015). Moreover, curcumin exhibits anti‐cancer properties on LC via various mechanisms, including suppression of cell growth, apoptosis induction, modifications to epigenetics, and control of microRNA expression (Kumar et al. 2016). Curcumin inhibits LC cell proliferation through cell cycle arrest, apoptosis, autophagy, and ROS‐mediated stress.

### 5.3 Epigallocatechin Gallate

Epigallocatechin gallate (EGCG) therapy effectively inhibits the proliferation of fusion gene‐ or EGFR‐driven LC cells, like H2228, in vitro and in vivo, and in cells carrying specific mutations (Honda et al. 2017). Additionally, EGCG effectively inhibits growth, reducing tumor angiogenesis and inhibiting HIF‐1 expression in xenograft tumors. In vitro studies suggested inhibition of EGFR, ALK, and phosphorylation, while in vivo effects were linked to Akt and ERK, suggesting tumor responsiveness (Honda et al. 2017). A study using cDNA expression array technology in human cancer found that EGCG significantly impacted the expression of multiple genes. Four genes increased, and twelve were downregulated. Downregulated genes included MAP kinase p38γ, CDC 25B/M‐phase inducer phosphatase 2, NF‐κB inducing kinase, DAPK1, SAP102, Rho B, T‐lymphoma invasion and metastasis inducing protein 1, Cdc42 GTPase‐activating protein, tyrosine‐protein kinase, and the EGFR gene. EGCG may control genes related to physiological functions (Okabe et al. 2001). H460 inhibited anchorage‐independent growth and proliferation in human NSCLC cells and mouse lung adenocarcinoma cells. EGCG administration increased miR‐210 expression in both cells. HRE in the miR‐210 promoter and HIF1 stabilization facilitate upregulation. HIF‐1α expression vector enhanced HRE‐luciferase's reaction to EGCG (Wang et al. 2011). EGCG regulates miRNA expression in a mouse LC model, controlling genes through miRNA‐mediated anticancer actions. In NNK‐induced mice, EGCG upregulated 12 miRNAs, affected 21 mRNAs, and targeted 26 genes (Liu, Cao, et al. 2014). Moreover, EGCG inhibits the cell cycle and inflammation (Singh, Shankar, and Srivastava 2011; Yang et al. 2009). EGCG administration in vivo significantly altered the expression of miR‐210, despite its absence in primary tumors (Liu, Cao, et al. 2014). Furthermore, EGCG and luteolin showed additive and synergistic apoptotic and growth‐inhibiting properties in LC models (Amin et al. 2010). EGCG inhibits LC growth by blocking EGFR/ALK signaling, decreasing angiogenesis, modulating gene expression, and inducing apoptosis in vitro and in vivo.

### 5.4 Quercetin

Quercetin, a polyphenol found in fruits and vegetables, has anticarcinogenic properties due to its ability to block enzymes that activate carcinogens and bind to proteins and cellular receptors (Canivenc‐Lavier et al. 1996; Shih et al. 2000). Additionally, quercetin has anti‐proliferative and pro‐apoptotic effects against LC (Rauf et al. 2018). Quercetin causes cytotoxicity and apoptosis in NSCLC cells in humans. It prevents cell division, terminates the G2/M phase, and triggers apoptosis through increased phospho‐p53, cdc2, survivin, cyclin B1, total p53, and p21 proteins, causing aberrant chromosomal segregation (Kuo et al. 2004). Furthermore, quercetin‐induced apoptosis is triggered by the activation of the MEK–ERK pathway. It inactivates Akt and increases phosphorylation of MEK, ERK, c‐Jun, and JNK (Nguyen et al. 2004). Quercetin treatment in H460 cells upregulated genes related to cell cycle arrest, TNFR1, FAS, TRAILR, JNK, IL1 receptor, and death pathways, while suppressing those related to cell proliferation and survival (Youn et al. 2013). Moreover, quercetin inhibits MCT1, a key anti‐invasive and anti‐metastatic agent in LC cells, by deconstructing microfilaments, preventing aurora B kinase activation, and preventing NSCLC cell movement and invasion (Izumi et al. 2011; Klimaszewska‐Wiśniewska et al. 2017; Xingyu et al. 2016). Quercetin reduced NSCLC cell migration and bone metastases in vitro, downregulating mesenchymal markers N‐cadherin and vimentin and upregulating epithelial markers E‐cadherin in living organisms. It blocks the Akt pathway, dependent on Snails, to prevent EMT, and the Snail‐independent ADAM9 pathway by rearranging F‐actin‐containing microfilament bundles (Chang et al. 2017). A study investigated the impact of five phytochemicals on LC cell migration and invasion caused by NiCl2. Quercetin, chrysin, and apigenin were found to be the most potent, preventing cell migration and invasion by downregulating the TLR4/NF‐κB signaling pathway (Wu et al. 2018). Quercetin and trichostatin A treatment of A549 cells expressing wild‐type p53 leads to growth arrest and mitochondrial death, indicating possible epigenetic processes (Chan et al. 2013). Quercetin inhibits LC proliferation, induces apoptosis, and arrests the cell cycle through pathways such as MEK–ERK, Akt, and p53 activation.

### 5.5 Luteolin

Luteolin, also known as 3′,4′,5,7‐tetrahydroxyflavone, has been found to possess anti‐inflammatory, anti‐cancer, and antioxidant properties. It has anti‐tumorigenic properties using a widely utilized LC cell line. It also caused G1 phase cell cycle arrest and death, which stopped A549 cells from growing. It inhibited cell migration and stress fiber assembly in A549 cells (Zhao et al. 2011). A study found luteolin's cytotoxic and pro‐apoptotic effects on the human NSCLC cell line A549. It inhibited cell cycle arrest and induced apoptosis in A549 cells, blocking NF‐κB translocation and activating JNK (Cai et al. 2011). Luteolin inhibits tumor growth in LC cells by triggering apoptosis and autophagy in gliomas through MAPK activation. It suppresses LIMK1 kinase activity, causes apoptosis, and reduces cell cycle arrest in LC cells. LIMK1 down‐regulation and its interaction with cofilin are key mechanisms for its anticancer effects, proving its potential in LC clinical trials (Zhang et al. 2021). Additionally, luteolin significantly decreased AIM2 expression, suppressing its activation, preventing EMT in NSCLC. Its anticancer effects were dependent on AIM2, and downregulating AIM2 may be a useful strategy for treating NSCLC (Yu et al. 2019). Furthermore, luteolin pretreatment prevented TGF‐β1‐induced morphological changes and downregulation of A549 LC cells' E‐cadherin. It reduced the activation of the PI3K–Akt–IκBa–NF‐κB–Snail pathway, causing E‐cadherin to decrease (Chen et al. 2013). Luteolin inhibits LC cell growth through apoptosis induction, autophagy activation, G1 cell cycle arrest, and by blocking migration and stress fiber formation.

### 5.6 Genistein

Genistein, an isoflavone found in soybeans, has been found to have anti‐tumor properties, including cell cycle arrest, apoptosis, and deactivation of signaling pathways in human cancer cells. A study on the SCLC cell line H446 found that genistein reduced cell proliferation, apoptosis, and G2/M phase cell cycle arrest, and strengthened cisplatin's anti‐proliferative action (Tian et al. 2014). A study investigated the potential anti‐cancer impact of genistein on A549 human LC cells. Genistein administration significantly inhibited cell proliferation, increased cell apoptosis, and dose‐dependently activated caspase‐3/9. It decreased the expression of MET protein in A549 cells and increased the expression of microRNA‐27a (miR‐27a). This study highlights a link between miR‐27a‐mediated MET signaling and genistein's anti‐cancer action for the first time (Yang, Zang, et al. 2016). In a trial, cisplatin and genistein were combined to inhibit tumor growth and lower the dosages needed for NSCLC treatment. In vivo studies showed that combining cisplatin and genistein significantly inhibited tumor growth and suppressed phosphoinositide‐3 kinase and constitutive phosphorylation of AKT (Liu, Yan, et al. 2014). Another study investigated the effects of genistein on H460 NSCLC. It inhibited cell growth, altered gene expression, and caused apoptosis. It also induced apoptosis and modulated p21WAF1 protein expression. The manipulation of cell growth, cell death, and cell cycle regulatory molecules may be the cause of genistein's biological actions (Lian et al. 1998). Furthermore, a study investigates the impact of genistein on A549 cell apoptosis using real‐time techniques. It induces apoptosis dependent on time and concentration, and its action is decreased by low IMPDH2 expression. The highly expressed AKT1 prevents genistein from impacting LC cell viability and apoptosis (Xu et al. 2022). Genistein inhibits LC cell proliferation by inducing apoptosis, causing cell cycle arrest, activating caspases, and modulating signaling pathways like MET, PI3K–AKT, and p21.

## 6 Dietary Plant‐Derived Effects on LC

### 6.1 Fruits

Fruits have exceptional antioxidant qualities that may help reduce cancer risks due to their high polyphenol content. Numerous fruits and the primary bioactive components of those fruits have shown anticancer promise in a variety of biometric systems and animal models (Khan et al. 2007; Singh, Sharma, and Katiyar 2011). A study demonstrates that a higher intake of fruits and vegetables is associated with a lower risk of LC for both men and women across various countries. Low blood β‐carotene levels are predictive of a higher risk of LC. However, a randomized, placebo‐controlled clinical study found that men who took β‐carotene supplements had a higher incidence of LC and overall mortality rate. Diets high in fat, saturated fat, and cholesterol may raise the risk of LC (Ziegler et al. 1996). The Netherlands Cohort Study assessed the relationship between fruit and vegetable consumption and LC incidence. A food‐frequency questionnaire measured dietary intake, including age, sex, smoking history, education level, and family history. Results showed significant inverse relationships between vegetable groups and total vegetable consumption, with Brassica vegetables having the greatest impact. Fruits and vegetables had a stronger protective effect on current smokers and less pronounced effects on adenocarcinomas (Voorrips et al. 2000). High intake of fruits and vegetables, which are high in polyphenols and antioxidants, correlates with a decreased risk of LC, especially in smokers.

#### 6.1.1 Pomegranates

Ancient cultures used pomegranate fruit as a folk cure and nutrient‐dense natural product. Pomegranate fruit extract was found to decrease A549 cancer cell viability by inhibiting cell cycle proteins like cyclins D1, D2, E, PI3K, and MAPK. It demonstrated inhibitory effects on tumor growth in athymic nude mice (Khan et al. 2007). Oral pomegranate fruit extract consumption significantly reduced LC in mice by blocking signaling pathways in drinking water, potentially reducing lung angiogenesis and cell growth (Adhami et al. 2009). Pomegranate fruits contain punicalagin, a high molecular weight polyphenol crucial for their biological activities. The cytotoxic effects of punicalagin on A549 cells were demonstrated in in vitro studies (Kulkarni et al. 2007). Additionally, punicalagin demonstrated antioxidant effects by forming metal chelates and removing free radicals from the body. It reduced oxidative DNA damage (Aqil et al. 2012). A study investigated the anticancer effects of *Punica granatum* leaf extract on NSCLC cell lines A549, H1299, and mice Lewis lung carcinoma cell line LL/2 in vitro. It inhibited cell growth, induced apoptosis, and stopped cell cycle progression, impacting H1299 cell survival. It also inhibited H1299 cell motility and invasion, and decreased matrix metalloproteinase expression (Li et al. 2016). Pomegranate extracts inhibit LC cell proliferation, induce apoptosis, and arrest the cell cycle in NSCLC cells.

#### 6.1.2 Blueberries

The Ericaceae family, which includes various blueberries, is prominent for its nutritious fruit (Prior et al. 1998). A study found that rabbit eye blueberries, along with ursolic acid, amyrin, and 3‐O‐glucopyranoside, effectively inhibited the growth of human LC PC‐12 cells using GI50 values of 2163, 1002, and 1901 mg/mL, respectively (Ono et al. 2004). Blueberry anthocyanidins, including cyanidin, malvidin, peonidin, petunidin, and delphinidin, induce cell cycle arrest, apoptosis, and inhibit NSCLC cells (Kausar et al. 2012). Male Sprague–Dawley rats exposed to blueberry anthocyanins showed a significant reduction in radiation effects on healthy lung tissue (Liu et al. 2015). Blueberry anthocyanins protect cells from radiation therapy‐induced death by altering Caspase‐3, Bax, and Bcl‐2 expression and protein kinase R signaling pathways, reducing tumor volume in nude mice (Aqil et al. 2016). A549 cell growth was inhibited by blueberry extracts with anthocyanin concentrations of 50–100 g/mL (cell growth suppression of 60%–70%) (Aqil et al. 2016). A study showed blueberries have significant antioxidant properties and inhibit cell proliferation in A549 cells. It demonstrated promising anticancer properties in molecular docking experiments against CDK6 and AMPK protein kinases. It also showed strong cytotoxic effects on A549 cells and enriched PI3K/AKT1/STAT and p53 signaling pathways (Krishnamoorthy et al. 2024). Volatile extracts from blueberries, black raspberries, and blackberries were studied for their anti‐proliferative effects on A549 NSCLC cells. Using gas chromatography–mass spectrometry, three berry volatile extracts were applied to A549 cells for 12, 24, and 48 h. All three BVEs, including monoterpene, significantly triggered apoptosis and suppressed cell growth. The percentage of cells in the G0/G1 interphase rose with two‐fold diluted BVE treatments. These volatiles may affect LC (Gu et al. 2022). Blueberries and their bioactive compounds, such as anthocyanidins and volatile extracts, inhibit LC cell proliferation, induce apoptosis, and cause cell cycle arrest in NSCLC cells.

### 6.2 Vegetables

Vegetables contain bioactive phytochemicals like glucosinolate, isothiocyanate, and carotenoids, which are known to combat cancer and other chronic diseases (Zhang, Gan, et al. 2015). A study found that cruciferous vegetables, high in isothiocyanates, may prevent LC. Variations in the GST genes may alter the relationship between cruciferous vegetable consumption and LC risk. The study found that individuals with GSTM1 and GSTT1 double null genotypes exhibited the strongest negative correlation between their overall cruciferous vegetable intake and the risk of developing LC. The incidence of LC may be inversely and weakly correlated with cruciferous vegetable consumption (Lam et al. 2009). Recent large‐scale research and clinical trials on β‐carotene supplementation have raised doubts about the preventive effects of eating fruits and vegetables against LC. Six cohort studies and four case–control studies found a slight inverse relationship between green‐yellow vegetable consumption and LC risk (Wakai et al. 2011). Cruciferous and green‐yellow vegetables, containing bioactive compounds such as isothiocyanates and carotenoids, may lower the risk of LC.

#### 6.2.1 Cruciferous Vegetables

Cruciferous plants such as watercress, radish, and cabbage are widely consumed globally. Isothiocyanates and glucosinolates, compounds derived from these vegetables, have shown exceptional anticancer properties (Wu et al. 2009). Myrosinase hydrolyzes glucosinolates to produce isothiocyanate. In A549 LC cells, the main glucosinolate present in radish, 4‐Methylsulfinyl‐3‐butenyl isothiocyanate, markedly and dose‐dependently enhanced mitochondria‐mediated apoptosis. MTBITC increased caspase‐3 expression, which decreased the potential of the mitochondrial membrane and −9 and lowered the levels of Bcl‐xL/Bax and Bcl‐2/Bax (Wang, Wang, et al. 2016). Sulforaphene is found in abundance in radish seeds. The administration of sulforaphene to nude Balb/C mice receiving an LC xenograft effectively inhibited tumor growth. Sulforaphene effectively suppressed the PI3K/Akt signaling pathway in LC cells at dosages ranging from 10 to 40 mol/L, reducing PTEN gene expression and inhibiting Akt phosphorylation (Yang, Wang, et al. 2016). Radish leaf extracts can cause apoptosis in p53‐deleted human LC cells (H1299 and Calu‐6) to lessen their viability. The extract (H1264) did not affect cancer cells that express wild‐type p53 (A549) or those producing a p53 mutant lacking the C terminus (Baeka et al. 2017; Li et al. 2017). The natural conjugate 2‐phenethyl isothiocyanate (PEITC) effectively reduces metabolic activation caused by the tobacco carcinogen 4‐(methylnitrosamino)‐1‐(3‐pyridyl)‐1‐butanone (NNK) when administered at 10 mg to F344 rats and A/J mice (Yuan et al. 2016). Cruciferous vegetables induce apoptosis and inhibit LC cell growth.

#### 6.2.2 Bitter Melons

Chinese and Indian bitter melon types, *Momordica charantia* , have been found to exhibit antiproliferative properties in human LC cells. Chinese hot aqueous extraction demonstrated strong antiproliferative effects, enhancing ROS and caspase‐3/7 activity. Its antiproliferative impact is attributed to apoptosis caused by ROS‐mediated mitochondrial damage (Thiagarajan et al. 2019). *Momordica charantia* methanol extract, when applied to CL1‐0 LC cells, exhibits potent anticancer properties due to its ability to control caspases and mitochondria, leading to DNA fragmentation and nucleic acid condensation (Li et al. 2012). The administration of bitter melon extract at concentrations of 0.15, 0.3, 0.6, and 1.25 mg/mL may prevent the migration of CL1 cells by decreasing their activation and expression (Hsu et al. 2012). Momordica anti‐HIV protein and momorcharin significantly increased cell cycle arrest and cell death in A549 cancer cells, suppressing cell proliferation in a dose‐ and time‐dependent manner. After 48 h of therapy, DNA degradation in A549 cancer cells was observed (Fan et al. 2015). Bitter melon extracts inhibit LC cell proliferation by inducing ROS‐mediated apoptosis, mitochondrial damage, and DNA fragmentation.

#### 6.2.3 Perilla

Perilla‐derived methoxyflavanone, a methoxyflavanone derivative from *Perilla frutescens* , induces cellular senescence in A549 human adenocarcinoma cells, activating the p53‐p21 pathway and requiring p53 for its pro‐senescent action (Maeda et al. 2022). Additionally, perilla has strong anti‐proliferative effects on A549 cells (Lin et al. 2010). A study found that perilla leaf extract at doses of 87.5, 175, and 350 g/mL significantly reduced H1299 NSCLC cells' migration and adhesion (Kwak and Ju 2015). Methoxyflavanone showed anticancer properties in A549 LC cells by inhibiting cell growth and viability, and up‐regulating CDK inhibitors (Abd El‐Hafeez et al. 2018a). The combination of anti‐cancer tyrosine kinase medications ponatinib, dasatinib, bosutinib, and nilotinib effectively inhibited the growth of A549 cells in athymic nude mice (Abd El‐Hafeez et al. 2018b). Another study investigates the molecular processes of rosmarinic acid‐rich fraction (RA‐RF) from perilla seed meal and its anti‐OS, anti‐inflammatory, and anti‐metastasis effects in A549 cells exposed to particulate matter from forest fires (PMFF). Results show that PMFF increases ROS production, pro‐inflammatory cytokine expression, MMP‐9 activity, invasion, migration, and Akt phosphorylation. RA‐RF functions through c‐Jun, p‐65‐NF‐κB, and Akt signaling pathways, potentially preventing PM‐induced lung inflammation and cancer metastases (Pintha et al. 2021). Perilla extracts and methoxyflavanone inhibit LC cell proliferation, induce senescence through the p53–p21 pathway, and decrease migration and adhesion in NSCLC cells.

### 6.3 Spices

Spices, including turmeric, black cumin, ginger, garlic, saffron, black pepper, and chili pepper, have been used for thousands of years as traditional medicines and food flavorings. Their antioxidant, anti‐inflammatory, and immunomodulatory properties have been studied for their potential in preventing and treating various cancers (Zheng et al. 2016). Spices reduce oncological risks due to modern diets. These nutraceutical ingredients have a negative correlation with cancer incidence and have strong antioxidant activity, which helps alleviate metabolic disorders. Promising compounds include curcumin, limonene, and saffron. These bioactive components inhibit cytochrome P450, CYP 1A1, STAT‐3, and signal transducers, which are linked to various cancers (Butt et al. 2013). Clove, a spice and flavoring plant, has been studied as a potential source of cancer chemopreventive substances. In a study using strain A mice, clove aqueous infusion decreased the incidence of hyperplasia, dysplasia, and carcinoma in situ in lung carcinogenesis. Clove therapy decreased proliferative cells and increased apoptotic cells, downregulating anti‐apoptotic protein Bcl‐2 and pro‐apoptotic proteins p53 and Bax (Banerjee et al. 2006). Spices inhibit LC progression through antioxidant, anti‐inflammatory, and immunomodulatory effects.

#### 6.3.1 Turmeric

Turmeric, a popular Indian spice in Chinese traditional medicine, is the primary component of curry powder. Angiogenesis, metastasis, and invasion of cancer cells are all significantly impacted by MMPs. Curcumin inhibited A549 cancer cell migration and invasion by downregulating MMP‐2 expression through MEKK3, p‐ERK signaling pathways (Lin et al. 2009; Tsai et al. 2015). Curcumin activated a ROS‐mediated mitochondrial pathway, leading to the death of SCLC NCI‐H446 cells by releasing cytochrome c and activating caspase‐9 to caspase‐3 (Yang, Ma, et al. 2012). Furthermore, curcumin treatment decreased Bcl‐2 and Bcl‐xL levels in NCI‐H446 cells and increased production of Bax. Curcumin, at 15 mol/L, not only inhibited SCLC cell proliferation but also did not phosphorylate STAT3. SCLC cells showed simultaneous down‐regulation of invasive proteins like VEGF, MMP‐2, MMP‐7, and ICAM‐1, as well as cell division‐promoting proteins like Cyclin B1, Bcl‐xL, and Survivin (Yang, Liu, et al. 2012). Additionally, curcumin induced AMP‐activated protein kinase signaling pathway‐mediated cell autophagy in A549 cancer cells, accompanied by increased acetyl‐CoA carboxylase (Xiao et al. 2013). Curcumin reduced the migration and invasion of TGF‐1‐ or 801D LC cells, which are triggered by epidermal growth factor. It inhibits the Rac1/PAK1 signaling pathway and reduces MMP‐2 and MMP‐9 synthesis, hence preventing the migration and invasion of 801D LC cells (Chen et al. 2014). Moreover, curcumin suppresses cell growth and causes cell death in NSCLC cells. The process involves increasing the expression of miR‐192‐5p and decreasing the PI3K/Akt signaling pathway (Jin et al. 2015). Curcumin inhibits LC cell proliferation, migration, and invasion by downregulating MMPs, VEGF, and Cyclin B1.

#### 6.3.2 Ginger

Ginger rhizome has therapeutic benefits in traditional medicine, treating gastrointestinal disorders, rheumatism, arthritis, and metabolic and cardiovascular disorders, among other conditions (Aggarwal and Kunnumakkara 2009; Tuntiwechapikul et al. 2010). Additionally, ginger contains several bioactive substances with antioxidant, anti‐inflammatory, or anticancer properties, including 6‐gingerol, zerumbone, and 6‐shogaol (Sang et al. 2009). Ginger aqueous extract breaks A549 cancer cells' microtubules, degrades their structural integrity, and causes cell death at various dosages (Choudhury et al. 2010). Furthermore, ginger reduces cancer‐specific molecular targets, c‐Myc, and human telomerase reverse transcriptase expression in A549 cancer cells (Tuntiwechapikul et al. 2010). After a 96‐h treatment with 80 g/mL ginger, cancer cells decreased to 26%. It may cause apoptosis, increase p53 expression, and decrease Bcl‐2 protein (Hessien et al. 2012). Zerumbone is a cyclic sesquiterpene that is isolated from ginger. Female A/J mice fed zerumbone at doses of 100, 250, and 500 ppm significantly reduced lung adenoma diversity and prevented lung carcinogenesis by altering NF‐κB and heme oxygenase‐1 expression (Kim et al. 2009). The exposure to zerumbone significantly reduced the viability of A549 and H460 NSCLC cells, respectively. Cancer cells undergo apoptosis when caspases 9 and 3 are activated, and mitochondrial cytochrome C is released (Hu et al. 2014). 6‐shogaol inhibits the abnormal EGFR signaling cascade proliferation, induces cell cycle arrest, and causes death. Additionally, 6‐shogaol, when administered in small amounts, effectively inhibited the phosphorylation of cyclin D1/3 and STAT3 in NCI‐H1650 LC cells. Furthermore, 6‐shogaol inhibited the growth of tumor cells in nude mice bearing an NCI‐H1650 tumor cell xenograft (Kim et al. 2014). Ginger inhibits LC cell proliferation, induces apoptosis via p53/Bcl‐2 modulation and caspase activation, and disrupts microtubules and EGFR signaling.

#### 6.3.3 Garlic

Garlic is used to treat various human malignancies due to its organosulfur compounds (Zhang et al. 2016). Allicin negatively impacts cancer cell line A549 at concentrations ranging from 0.0047 to 1.2 mM in terms of viability and proliferation (Gruhlke et al. 2016). Dialyl disulfide, diallyl trisulfide, and diallyl sulfide are three primary oil‐soluble allyl sulfides found in garlic, which significantly enhance Bax translocation, cytochrome release, and caspase‐3 and caspase‐9 activation. These changes led to cell death by activating the Bax‐triggered mitochondrial pathway (Nagaraj et al. 2010). Dialyl trisulfide effectively inhibits human LC cell line survival by causing G2/M phase cell cycle arrest and death, possibly due to down‐regulation of CDK1 and buildup of phospho‐Tyr 15 (Xiao et al. 2009). Furthermore, dialyl trisulfide upregulates pro‐apoptotic proteins, leading to cell death at various doses. Garlic's ajoene, an organosulfur chemical, has strong anticancer effects, causing apoptosis in LC cell lines, involving ROS‐mediated signaling (Wang, Sun, et al. 2016). S‐allylmercaptocysteine effectively combats carcinogenesis by controlling the cell cycle, reducing ROS production, protecting DNA, activating SOD, and blocking NF‐κB activity (Wang, Sun, et al. 2016). Garlic inhibits LC cell proliferation, induces G2/M cell cycle arrest, and triggers apoptosis via Bax‐cytochrome c‐caspase and ROS‐mediated mitochondrial pathways.

## 7 Safety Profile, Off‐Target Effects, and Drug–Drug Interactions

Combination therapies for NSCLC present challenges in design and testing. A large dataset analyzing 81 cell lines with over 5000 targeted agent combinations reveals significant variability in tumor responses. While combinations rarely exceed the efficacy of single therapies, co‐targeting closely related genes can enhance drug activity, indicating a potential for more effective combinations. The context‐specific nature of these combinatorial effects allows for tumor specificity (Nair et al. 2023). The increasing death rate and low five‐year survival for NSCLC highlight the urgent need for effective treatments. ICI demonstrates potential with significant results, but the risk of immune overstimulation is a serious concern. The study demonstrates NP‐based ICIs and analyzes 38 trials involving 16,781 participants. Mixed effects analyses revealed that ICI treatment is more effective and associated with lower mortality compared to traditional therapies. ICI is recommended as a first‐line treatment option due to its proven effectiveness and safety (Majernikova 2024). A recent network meta‐analysis involving 22,178 patients from 38 RCTs assessed the risk of adverse events across various ICI‐based regimens. Chemotherapy‐containing treatments had a higher likelihood of treatment‐related AEs compared to ICI‐based regimens. For grade 1–5 irAEs, the highest risks were associated with dual ICIs + chemotherapy (50.5% probability of being the most harmful), followed by dual ICI therapy (47.2%), ICI monotherapy (80.0%), ICI monotherapy + chemotherapy (98.0%), and chemotherapy (100.0%). Similar trends were observed in grade 3–5 irAEs. ICI monotherapy combined with chemotherapy is preferred due to its immune‐related safety profiles, indicating that the safety of ICI‐based therapies varies according to the type and severity of adverse events (Yan et al. 2021). Vasculogenic mimicry contributes to radiation resistance in LC. In this study, celecoxib, a COX‐2 inhibitor, was evaluated for its radiosensitizing effects on NSCLC and its ability to control vasculogenic mimicry through off‐target actions. Experiments with BALB/c and C57 mice revealed that celecoxib treatment significantly reduced vasculogenic mimicry production induced by irradiation. Celecoxib reduces vasculogenic mimicry development by lowering the expression and activity of aminopeptidase N and integrin alpha‐V, as identified through molecular docking of four potential off‐targets. Celecoxib effectively inhibits vasculogenic mimicry in NSCLC while blocking these newly identified targets, independent of COX‐2 inhibition (Niu et al. 2021). Targetable changes in cancer form new drug discovery opportunities, but solubilizing medications in dimethyl sulfoxide is often required for pre‐clinical testing. This study evaluated the off‐target effects of dimethyl sulfoxide on signaling networks and pharmacological targets in multiple cell lines utilized in pre‐clinical testing. Three dimethyl sulfoxide concentrations were tested on eight NSCLC cell lines, with a reverse phase protein array measuring the expression and activation of 187 proteins. Results showed significant variations in protein levels dependent on dimethyl sulfoxide dose, exposure duration, and specific cell lines, particularly at the highest concentration. Ultra‐low concentrations of dimethyl sulfoxide can significantly influence targetable signaling proteins and demonstrate caution in pre‐clinical drug screening to consider off‐target effects (Baldelli et al. 2021). In a study involving advanced NSCLC patients treated with EGFR tyrosine kinase inhibitors, a software was utilized to identify drug–drug interactions. A total of 20 clinically significant drug–drug interactions were identified, impacting 14 patients, mainly involving interactions with calcium antagonists, SSRIs, antipsychotics, antiepileptics, and H2‐receptor antagonists. Notably, progression‐free survival was negatively correlated with statin use. This study assesses the clinical implications of drug–drug interactions and their effects on the efficacy of EGFR‐tyrosine kinase inhibitors in advanced NSCLC (Occhipinti et al. 2021).

## 8 Molecular Pathways in LC

### 8.1 Epigenetic Modifications in the Regulation of Genes

LC exhibits abnormal epigenetic landscapes, with histopathological subgroups associated with hypomethylation of oncogenes and retrotransposons, as well as abnormalities in histone proteins. Epigenetic changes in microRNA expression influence the development of LC. These markers can be used for risk assessment, prognosis, disease monitoring, and early diagnosis (Brzeziańska et al. 2013). Additionally, LC is caused by genetic and epigenetic alterations, with epigenetic modifications like DNA methylation and histone acetylation being reversible with medications. This study combined genome‐wide expression profiling with pharmacologic suppression of these changes in NSCLC cell lines to identify new disease biomarkers. Three genes upregulated by treatments were downregulated and hypermethylated, while histone deacetylation dominated gene regulation (Zhong et al. 2007). The epigenetic mechanisms of gene control significantly influence the preservation of stem cells and the imprinting of the second female X chromosome in healthy conditions. Early epigenetic changes occur during the development of LC (Belinsky et al. 2002; Herman and Baylin 2003; McCabe et al. 2009). Genes involved in LC's Wnt‐ and Ras‐signaling pathways, apoptosis inducers like DAPK, and DNA repair genes like MGMT and hMLH‐1 are involved. The inhibition of cancer‐causing chemicals in human bronchial epithelial cells was achieved through siRNA silencing using DNMT1 siRNA (Damiani et al. 2008), and a combination of DNMT and HDAC pharmacologic suppression stopped the growth of mice with LC (Belinsky et al. 2003). A study found the effect of resveratrol on ZFP36 expression in A549 LC cells. It increased ZFP36 expression while decreasing the levels of its target genes. Additionally, it also led to the demethylation of the ZFP36 promoter and inhibited DNA (cytosine‐5) methyltransferase 1 production. It exerts anticancer effects through epigenetic regulation of ZFP36 in NSCLC (Fudhaili et al. 2019). Curcumin enhances the sensitivity of NSCLC cells to crizotinib and regulates miR‐142‐5p expression. NSCLC cell lines and tissues show significant downregulation of miR‐142‐5p, and curcumin potentially increases crizotinib's cytotoxic effects by restoring miR‐142‐5p levels. Furthermore, curcumin therapy reduces the activity of DNA methylation enzymes (DNMT1, DNMT3A, DNMT3B) and, like miR‐142‐5p overexpression, intensifies crizotinib‐induced cell death while inhibiting autophagy in A549 and H460 cells. The miR‐142‐5p inhibits autophagy by targeting Ulk1, and its overexpression negates the cytotoxic effects of crizotinib mediated through Ulk1 inhibition. Curcumin inhibits autophagy via miR‐142‐5p regulation, increasing NSCLC cells' susceptibility to crizotinib (He et al. 2022). LC is characterized by abnormal epigenetic changes such as DNA methylation, histone modifications, and altered microRNA expression, which impact oncogenes, tumor suppressors, and signaling pathways.

### 8.2 EGFR–RAS–MAPK Pathway

The prognosis for advanced NSCLC patients with EGFR‐activating mutations remains unfavorable due to intrinsic or acquired resistance. Clinical outcomes have improved with the introduction of EGFR‐TKIs. The molecular heterogeneity of LC has multiple implications, including secondary mutations, alternative signaling, abnormal downstream pathways, and apoptosis impairment (Morgillo et al. 2016). A study analyzed p65BTK expression in non‐smoking EGFR‐wt adenocarcinomas and 382 NSCLC patients. The results showed that p65BTK was overexpressed in non‐smoking patients with EGFR‐wild type adenocarcinomas and was maintained at the metastatic site. BTK‐TKIs significantly reduced cell proliferation and clonogenicity, and were more successful than EGFR‐TKIs in reducing cancer cell viability. Non‐toxic dosages of BTK‐TKIs re‐sensitized drug‐resistant NSCLC cell lines to target and standard‐of‐care chemotherapy. BTK‐TKIs in combination with standard‐of‐care chemotherapy and EGFR‐targeted therapy may form new clinical trials in NSCLC (Giordano et al. 2019). Bone morphogenetic proteins (BMPs) are crucial in controlling cell division, migration, proliferation, and programmed cell death. They interact with the Erk and MAPK pathways and have been linked to numerous tumors. BMP3b and BMP6 may be epigenetically inactivated in NSCLC and cell lines. The coactivation of BMP3b and BMP6 is linked to k‐ras mutations in LC. Lung carcinogenesis involves the simultaneous inactivation of BMP and activation of Ras signaling pathways (Kraunz et al. 2005). EGFR‐RAS‐MAPK signaling contributes to NSCLC progression, therapy resistance, and poor prognosis, with molecular heterogeneity affecting mutations, downstream pathways, and apoptosis.

### 8.3 PI3Kinase–AKT–mTOR Pathway

The PI3K signaling pathway is crucial for cellular processes like apoptosis, translation, metabolism, and angiogenesis. Recent research on PI3K signaling pathways and NSCLC has increased, with inhibitors of different PI3K pathway components being explored (Jiang et al. 2022). Akt/mTOR is overexpressed in LC. Natural or synthetic compounds can be utilized to treat LC by targeting the downregulation of this pathway. Alkaloids and flavonoids can be used to treat LC (Ghareghomi et al. 2021). The PI3K/AKT/mTOR pathway, responsible for cell growth and proliferation, is frequently dysregulated in cancer, leading to increased aggressiveness and worse prognosis in NSCLC. Developing selective inhibitors targeting this system is challenging due to resistance. Co‐targeting potential mediators may enhance the response to PI3K inhibition, potentially overcoming immunological evasion, chemoresistance, and radioresistance (Heavey et al. 2014). A study investigated the impact of AB23A on human NSCLC cells' survival and apoptosis. It significantly reduced A549 cells' viability, increased apoptosis, and stopped the cell cycle in the G1 phase. It also inhibited cell invasion and migration and decreased phosphorylated AKT, PI3K, and mTOR protein levels (Liu et al. 2019). The PI3K‐AKT–mTOR pathway regulates cell growth, proliferation, and survival, and its dysregulation promotes NSCLC aggressiveness and therapy resistance.

### 8.4 Wnt‐Signaling Pathway

Wnt/β‐catenin changes are common in human cancers, with overexpression of certain Wnt‐pathway components linked to poor prognosis in NSCLC. The downregulation of Wnt inhibitors, such as hypermethylation, is prevalent in NSCLC tumor cell lines and resected samples. Wnt signaling significantly impacts NSCLC carcinogenesis, prognosis, and treatment resistance. The loss of Wnt signaling inhibitors is a significant issue, primarily due to promoter hypermethylation (Stewart 2014). Additionally, the Wnt/β‐catenin pathway is linked to resistance and poor prognosis in NSCLC. SNPs for AXIN2, Wnt‐5B, CXXC4, and WIF‐1 were associated with survival. Patients with unfavorable genotypes had median survivals of 19.7, 15.6, and 10.7 months, respectively. The study explores Wnt inhibitors in advanced NSCLC due to low statistical power, patient heterogeneity, or false‐positive data in cohorts (Stewart et al. 2014). The Wnt signaling pathway is crucial for tissue and organ development, controlling apoptosis, genetic stability, migration, differentiation, and cell proliferation. Mutations can lead to diseases like cancer, with high activation of the Wnt/β‐catenin pathway being closely linked to LC (Zhang and Wang 2020). LC is influenced by dysregulation of Wnt signaling, which is controlled at various cellular levels. This dysregulation may lead to resistance to targeted therapy, chemotherapy, and radiation. MicroRNAs regulate Wnt signaling, and the Wnt pathway may provide LC indicators and treatment options. Wnt pathway inhibitors have shown efficacy in treating epithelial cancers (Yang, Chen, et al. 2016). Curcumin has effects on LC stem cells derived from A549 and H1299 cells. It reduced tumorsphere formation, lowered the number of CD133‐positive cells, decreased marker expression, suppressed proliferation, and induced apoptosis in LC stem cells. Additionally, it also inhibits the Sonic Hedgehog and Wnt/β‐catenin signaling pathways (Zhu et al. 2017). Resveratrol dose‐dependently increases PD‐L1 expression in LC cells, which is significant for inhibiting T‐cell‐mediated immune responses. The Wnt pathway is essential, with resveratrol enhancing SirT1 deacetylase to stabilize the transcription factor Snail. This leads to the disruption of the destruction complex and promotes β‐catenin/TCF binding to the PD‐L1 promoter. Resveratrol impacts anti‐tumor immunity by regulating PD‐L1 expression (Yang et al. 2021). Abnormal Wnt/β‐catenin signaling in NSCLC, often caused by hypermethylation of Wnt inhibitors, promotes tumor progression, poor prognosis, and therapy resistance.

### 8.5 DNA‐Adduct Formation

Tobacco smoke causes LC through the formation of lung promutagenic DNA adducts. LC frequently has p53 mutations, and exposure to carcinogens has been linked to these patterns in LC (Guinee et al. 1995; Kishimoto et al. 1992; Ryberg et al. 1994). A study in Taiwan found that LC patients had significantly higher levels of DNA adducts than non‐cancer controls. Smoking habits and cigarette consumption did not affect the levels of DNA adducts in LC and non‐cancer samples. DNA adduct levels were not related to genetic variants like GSTM1, CYP1A1, or GSTM1. High DNA adduct levels were 25 times more likely to develop LC than low levels. LC may develop due to increased susceptibility to DNA damage (Cheng et al. 2000). Additionally, a meta‐analysis of 22 studies found that DNA adducts, a biomarker for exposure to carcinogens in tobacco smoke, are significantly linked to an increased risk of LC. LC patients had higher levels of bronchial adducts than controls. The production of bronchial adducts was also found to be significantly correlated with inhalation exposure to volatile carcinogens in tobacco smoke (Ceppi et al. 2017). Another study comparing 5p15.33, 6p21.33, and 15q25.1 genotypes in 365 NSCLC cases and 440 controls found dose‐dependent correlations with LC risk. The risk allele of rs402710 (TERT‐CLPTM1L gene) was associated with higher amounts of bulky aromatic/hydrophobic DNA adducts, suggesting a possible link between the TERT‐CLPTM1L variation and bulky DNA adducts, potentially influencing LC susceptibility (Zienolddiny et al. 2009). Furthermore, a study investigated the role of environmental carcinogens in LC development in Taiwanese female nonsmokers. LC patients had significantly higher DNA adduct levels than nonsmokers, and females had higher quantities of DNA adducts. Genetic polymorphisms were not responsible for these differences. High LC death rate among nonsmokers may be partly due to women's increased susceptibility to DNA damage (Cheng et al. 2001). DNA adducts are linked to increased p53 mutations and a higher risk of LC.

## 9 Conclusions and Future Perspectives

LC, a major global cause of cancer‐related morbidity and mortality, is often limited by drug resistance, side effects, and poor prognosis. Targeted phytocompounds, which can alter various molecular signaling pathways like PI3K/Akt/mTOR, MAPK/ERK, NF‐κB, and STAT3, have become promising treatments for LC. These compounds demonstrate significant antiproliferative, pro‐apoptotic, anti‐inflammatory, and anti‐metastatic properties in both preclinical and clinical studies. Traditional medicines often target a single pathway, but these medicines have a multi‐targeted mechanism of action, setting them apart from traditional treatments. Furthermore, phytocompounds are promising for LC prevention and treatment due to their improved patient tolerance and decreased toxicity. Despite promising results, the variability in pharmacokinetics, bioavailability, and standardized formulations remains a significant obstacle in converting preclinical data into reliable clinical outcomes. Future research should focus on enhancing the stability, solubility, and bioavailability of phytocompound‐based medicines through structural modification, nanotechnology, and advanced drug delivery systems. To validate preclinical findings and establish effective dose regimes, well‐designed clinical trials with larger cohorts are needed. Furthermore, combining phytocompounds with existing immunotherapies or chemotherapeutic drugs may lead to synergistic effects, reducing resistance and improving patient outcomes. Precision medicine techniques, based on molecular profiling, can provide personalized phytocompound‐based treatments for LC patients with unique genetic and epigenetic changes. Investigating phytocompounds as chemopreventive agents in high‐risk groups has significant potential to reduce LC incidence. The use of phytocompounds as adjunctive treatments in LC treatment provides a safer, more efficient, and patient‐centered approach.

## Author Contributions

**Md. Rezaul Islam:** conceptualization, methodology, investigation, writing – original draft, writing – review and editing, resources, visualization. **Abdur Rauf:** conceptualization, investigation, methodology, visualization, resources, writing – original draft, writing – review and editing, supervision, funding acquisition, project administration. **Happy Akter:** writing‐original drafty, writing review and editing, resources, visualization. **Md. Ibrahim Khalil Al‐Imran:** methodology, investigation, writing – original draft, writing – review and editing, resources, visualization. **Md. Naeem Hossain Fakir:** writing original draft, resources, writing review and editing, visualization. **Gazi Kaifeara Thufa:** writing original draft, resources, writing review and editing, visualization. **Umme Habiba:** writing original draft, resources, writing review and editing, visualization. **Karjin Nahar Riya:** resources, writing review and editing, visualization. **Md. Mahfuzur Rahman:** resources, writing review and editing, visualization. **Md Sadique Hussain:** conceptualization, investigation, methodology, resources, writing – review and editing, visualization. **Hanan A. Ogaly:** resources, writing review and editing, visualization. **Abdullah S. M. Aljohani:** writing review and editing, visualization, resources. **Waleed Al Abdulmonem:** conceptualization, investigation, methodology, writing – review and editing, visualization. **Dorota Formanowicz:** conceptualization, investigation, methodology, visualization, writing – original draft, writing – review and editing, resources, supervision, project administration, funding acquisition. **Marcello Iriti:** conceptualization, investigation, writing – original draft, writing – review and editing, methodology, visualization, resources, supervision.

## Funding

This work was supported by the King Khalid University (RGP2/95/46).

## Conflicts of Interest

The authors declare no conflicts of interest.

## Data Availability

The data that support the findings of this study are available from the corresponding author upon reasonable request.
